# Diversity in optical coherence tomography normative databases: moving beyond race

**DOI:** 10.1186/s40942-020-0208-5

**Published:** 2020-03-05

**Authors:** Nihaal Mehta, Nadia K. Waheed

**Affiliations:** 1 The Warren Alpert Medical School of Brown University, Providence, RI USA; 2 Department of Ophthalmology, New England Eye Center, Tufts Medical Center, 800 Washington Street, Box 450, Boston, MA 02111 USA

**Keywords:** Optical coherence tomography, Normative database, Race, Race-based medicine, Diversity

## Abstract

Normative databases of optical coherence tomography (OCT) metrics, such as retinal nerve fiber layer (RNFL) and macular thickness, are critical to clinical use of OCT imaging. In order to accurately represent the range of normal variation in patient populations, these normative databases must themselves be adequately diverse. Thus far, diversity in OCT normative databases has largely been defined as racial diversity. However, this has largely been based on self-reported “race,” which is inconsistent and generally not scientifically rigorous as a form of categorization. Moreover, there is a great deal of variation even within any single racial group, suggesting that other drivers of variation, such as geography or socioeconomic status, may be more important metrics for diversity. Finally, race itself is a proxy for the biological variation that must be represented in such samples, and as such racial diversity does not itself inherently equate to adequate biologic diversity. As clinical use of OCT continues to grow, including to international settings, it is increasingly important that normative databases built into OCT systems accurately represent the populations to which they are applied. Race is not an ideal sole or even primary means of assessing sample diversity in this context. In future normative OCT database construction, other forms of diversity should be considered.

## Normative OCT data

**Fig. 1:**
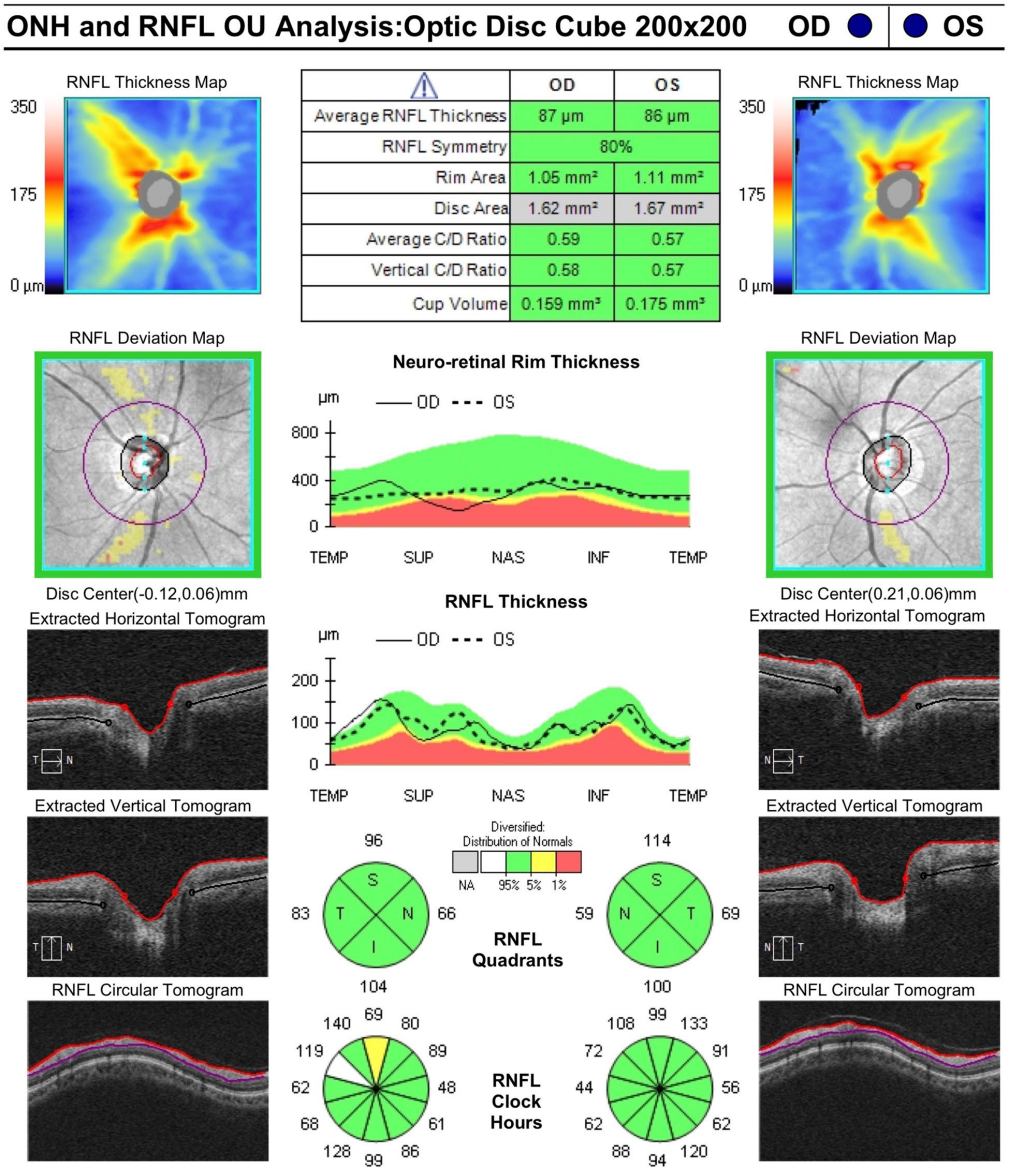
Optic nerve head and retinal nerve fiber layer analysis report. Normative databases are critical to the determination of normal versus abnormal measurements

Optical coherence tomography (OCT) was invented only three decades ago. In the years since, it has rapidly gained widespread adoption and become indispensable in the practice of ophthalmology. Critical to this success has been the ability of analytic software to automatically and objectively produce clinically meaningful measurements, such as the thickness of the peripapillary retinal nerve fiber layer (RNFL) in tracking glaucoma progression or the total retinal layer in assessment of macular diseases. In order to accurately interpret these values, databases of measurements drawn from normal eyes needed to be constructed. These databases are now in-built into all major commercial OCT systems, allowing for the widely used color-coded reports and progression maps that assist in rapid recognition and tracking of pathology (Fig. 1).

Such normative databases must adequately represent the normal range of population-level variation in the measurement under study in order for accurate conclusions to be drawn (i.e., whether measurements from a patient’s eye can be considered “normal”). This is increasingly the case as the global adoption of OCT systems expands, and, consequently, so does the size and variation of the patient population. In addition, novel OCT technologies, including swept source (SS)-OCT, will allow for new realms of quantification, such as measurements of the choriocapillaris, and will necessitate construction of entirely new normative databases. Consequently, it is critical to ensure that current and future OCT normative databases are adequately representative of the increasingly varied populations to which they are applied.

## Diversity in normative OCT databases

To this end, ensuring diversity in the pool of subjects used to construct normative databases is imperative, as this increases the likelihood that the sample will be adequately representative. “Diversity,” however, can be defined in many ways, including but not limited to age, gender, geographic area of origin, and socioeconomic status. Thus far, race-based categorization has played a major role in assessing sample population diversity for the purposes of normative OCT database construction. The earliest normative studies for macular thickness were completed using the Stratus time domain (TD)-OCT, with the Stratus normative database ultimately constructed after a study of about 350 eyes at several centers in the United States [1]. Similarly, the Carl Zeiss Cirrus normative databases for macular and RNFL thicknesses were based on data from a 7-site study enrolling just upwards of 280 subjects [2]. The Optovue RTVue Avanti system incorporated a larger normative database of about 640 eyes from 11 sites worldwide [3]. And the Heidelburg Spectralis RNFL thickness database included 330 eyes enrolled in Canada, the United States, and Germany [4]. All of these studies attempted to enroll diverse study populations, and report on their cohorts by age, gender, and conventional racial categorizations, such as Caucasian/White, African-American/Black, Hispanic, and Asian. Diversity has thus been defined in large part by the *racial* makeup of the sample population.

## A race-based definition of diversity

Although such studies were important starting points in the early stages of OCT research, it would be beneficial to carefully assess whether a largely race-based means of measuring sample diversity is the best approach. In the United States, there is a long history of race-based medicine and a growing body of scholarship that has critically analyzed its legacy and impact. This work suggests several reasons for caution in relying on race as a primary measure of diversity in OCT normative databases. First, self-reported race—as is used in the majority of normative OCT studies—is largely subject to sociocultural factors and arguably inadequately accurate or scientific. Lundy Braun has pointed out “the variability in methods of racial classification in different countries and how these classification systems change over time,” citing, for example, that someone who self-identifies as “coloured” in South Africa may instead be classified as “black” in the United States: a difference borne of the unique histories of each country, not any scientific or biological reality [5]. In addition, patterns of racial variability in biomarkers are contextual and can vary based on, for example, geography, suggesting that other factors apart from race are more proximate drivers of variation; in the words of Troy Duster, there is a “complex feedback loop and interaction effect between phenotype and social practices related to that phenotype” [6]. The overarching issue with a race-based approach to medicine, as Dorothy Roberts has highlighted, is that race is a fundamentally social categorization that is often inaccurately seen in medical contexts as adequately representative of biological or even genetic variation, easily resulting in scientifically flawed conclusions [7]. The fact that there exists far greater genetic variation within races than between was shown almost two decades ago [8]. Consequently, racial diversity may not correspond to the actual physiological variation in RNFL or macular thickness needed for a normative OCT database, which would be better ensured through socioeconomic, environmental, and geographic variation in study populations. A race-based approach could thus easily lead to a false sense that data are adequately diverse when, in fact, they lack other critical and arguably more important forms of representation.

## Moving beyond race-based diversity

Future normative OCT research studies should expand their definition of “diversity” beyond one that is race-based and instead more carefully assess and report other metrics of diversity in their sample populations, such as socioeconomic and geographic origin, and ensure their samples are adequately varied across these measures. For example, while the body of international OCT research is rapidly growing, several of the studies that generated widely used normative databases were based in the United States (perhaps as a result, the race groups used in these studies are largely based on American racial categorizations). Drawing from global studies would be a first step in expanding the scope and generalizability of normative data. A recent American-based study that assessed RNFL thickness categorized their subjects by race as Caucasian, Hispanic, African American, and Asian [9]. However, a major Singapore-based study of more than 8000 eyes found significant differences in RNFL thickness between Chinese-, Malay-, and Indian-origin participants, with “origin” defined by country of birth [10]. As the authors point out, these are all groups that would be considered “Asian” using conventional race-based definitions. As the example provided by these two studies illustrates, “Asian” (or any racial categorization) contains a great deal of variation within it and is likely too imprecise and poorly representative of a grouping for scientific and medical purposes.

## Conclusions

With increased adoption and further technological advances, OCT will no doubt continue to transform the practice of ophthalmology. It is important to ensure that the social elements that inevitably factor into the creation and interpretation of OCT data are critically assessed. In the case of normative databases, chief among these tasks is recognizing the shortcomings of race-based categorization. The laudable goal of effectively serving diverse patient populations would be better met in future normative research by incorporating other metrics of variation and not by assuming racial categorization of subjects automatically ensures diversity.

## Data Availability

Not applicable.

## Abbreviations

OCT: Optical coherence tomography
RNFL: Retinal nerve fiber layer
SS: Swept-source
TD: Time-domain

## References

[1] Retinal Physician—normative databases in SD-OCT: a status report. Retin Physician. 2010. https://www.retinalphysician.com/issues/2010/april-2010/normative-databases-in-sd-oct-a-status-report. Accessed 31 Jan 2020.

[2] Carl Zeiss Meditec, Inc. 510(k) summary: cirrus HD-OCT with retinal nerve fiber layer and macular normative databases. Food Drug Adm. https://www.accessdata.fda.gov/cdrh_docs/pdf8/K083291.pdf. Accessed 31 Jan 2020.

[3] Optovue, Inc. 510(k) summary: RTVue with normative database. Food Drug Adm. https://www.accessdata.fda.gov/cdrh_docs/pdf10/K101505.pdf. Accessed 31 Jan 2020.

[4] Heidelberg Engineering. 510(k) summary: spectralis HRA+OCT and variants. Food Drug Adm. https://www.accessdata.fda.gov/cdrh_docs/pdf15/K152205.pdf. Accessed 31 Jan 2020.

[5] Braun L (2002). Race, ethnicity, and health: can genetics explain disparities?. Perspect Biol Med.

[6] Duster T (2005). Medicine. Race and reification in science. Science.

[7] Roberts DE (2011). What’s wrong with race-based medicine?: genes, drugs, and health disparities. Minn J Law Sci Technol..

[8] Rosenberg NA, Pritchard JK, Weber JL, Cann HM, Kidd KK, Zhivotovsky LA (2002). Genetic structure of human populations. Science.

[9] Poon LY, Antar H, Tsikata E, Guo R, Papadogeorgou G, Freeman M (2018). Effects of age, race, and ethnicity on the optic nerve and peripapillary region using spectral-domain OCT 3D volume scans. Transl Vis Sci Technol..

[10] Ho H, Tham Y-C, Chee ML, Shi Y, Tan NYQ, Wong K-H (2019). Retinal nerve fiber layer thickness in a multiethnic normal Asian population: the Singapore epidemiology of eye diseases study. Ophthalmology.

